# Annotations

**Published:** 1898-12-31

**Authors:** 


### Annotations.

### Another Specialism: The Consulting Cyclist.

The growing use of the bicycle and its frequent prescription as a means to health suggests, as a possibility which, in fact, is already not far from its accomplishment, the evolution of a new kind of medical specialist, the consulting cyclist, who will devote himself to giving medical and practical advice as to all that concerns the use of the machine; whether to ride or not; what sort of a machine to ride; at what pace to ride; how the saddle is to be adjusted; where the handles are to be set; how the machine should be geared, &c.; all being things which differ for each individual. For in truth the fitting of the machine to the individual is a matter of no small nicety, and is one in regard to which the advice of a medical man knowing in such matters is of considerable importance. Many a doctor recommends the use of the cycle who is himself no cyclist, just as he may recommend hydropathic treatment, although he may know but little about the various combinations of bath treatment which will be found of greatest use at the particular spa resorted to. In stating the broad fact that cycling will do good he is acting within the range of his own knowledge and experience, but when he is asked about speed and gears and lengths of run, unless he is a cyclist as well as a medical man, he is apt to find himself at sea, and so is tempted either to deal with these matters "on general principles," or to refer his patient to the dealer. But surely the decision as to all the details of bicycling, especially when bicycling is undertaken for health purposes, is a medical affair, and is also one which may very properly be made a specialty. A girl will not be then merely told to ride a bicycle, but she will, under proper medical supervision, be put through a course of bicycling—a very different affair—and an elderly man who is ordered a machine will be told and shown what he may do and how far he may go in his search for youth without straining his heart and losing even such youth as is still left to him.

### The "Attractiveness" of the Workhouse.

A few years ago we had a great outcry against outdoor relief, and the duty of conscientious Guardians was felt to be to prevent people thinking that a weekly dole was to be had from the parish as a convenient addition to their ordinary income. It is hardly necessary now to point out how dangerous the loose granting of outdoor relief may be, and how it may pauperise the recipients without benefiting them, even in a material sense. The cry then was to impose unflinchingly the "workhouse test." This has been very generally done, and now we have a new danger—that the workhouse is being made too attractive. The same false sentimentality that has been restrained from handing out other people's half-crowns with the lavishness that feels itself to be, and passes for, generosity, now finds a vent in adding dainties to the paupers' bill-of-fare, and generally trying to make them as comfortable as possible, at the ratepayers' expense. The paper which Mr. Bailward, of Bethnal Green, read at the recent conference of poor-law authorities sets before us a danger which should put everyone who has the welfare of the industrious poor on the alert at once. When it comes to giving, as Mr. Bailward says was done in one workhouse, a daily diet of breakfast, lunch, dinner, an early cup of tea and three ounces of cake, tea again with bread and butter and more cake, and finally supper, we feel that, instead of the poor being deterred from entering the workhouse, they are absolutely tempted to do so; and only a very confirmed craving for drunkenness and dirt will keep them out. The vicious poor who desire these things may stay out, but the respectable poor will hasten to go in. Even in the case of children, where probably the temptation is most strong, it is necessary to be on one's guard against too great kindness. It is true that it is not the children's fault that they are paupers, but that of their parents. But if to them the workhouse is made too attractive, if they receive there anything beyond the absolute requisites for health of body and mental training, will they have the feeling which it is desirable they should have against returning to the workhouse in later life? Will they have the unwillingness which it is desirable they should have against their children being brought up in the workhouse as they were themselves? There are not wanting signs that the old horror of the workhouse—extreme, no doubt, and perhaps based on too great harshness in the treatment of paupers in former days, but wholesome in its effects on those outside—is beginning to fail. What are we to think of the case mentioned by Mr. Lawson, of Fulham, of the wives of working men going into the workhouse for their confinement to save the incidental expenses? Is not this a step towards that millennium of the lazy, when everyone shall be supported by the State? How fatal that would be to us as individuals and as a nation it is needless to detail. But shall we take warning in time?

### The Magistrate and the Coroner.

Medical men so often suffer at the hands of coroners that we fancy not a few will have enjoyed the newspaper accounts of the discomfiture of one of the best known of the London coroners in a case lately tried at the South-Western Police Court by Mr. Plowden. The point which led to the strictures of the magistrate was the irregularity of the proceedings in the coroner's court, and we need hardly say that most of the annoyances which medical men have to put up with at inquests are due purely to the irregularity with which such inquiries are too often conducted, for all comment derogatory to a witness and all attribution of blame, except such as is contained in the verdict, is absolutely irregular. In the case before Mr. Plowden—a charge of perjury against a medical man, for which there was shown to have been no excuse—it appeared that the coroner had asked the opinion of the jury before the whole of the evidence had been given! This they gave, thus leaving it open to counsel engaged to maintain that this was their verdict. But when the evidence was finished they reversed this opinion! Now it may be true, as the coroner in question is reported to have said, that this plan "saved time," and saved him "a lot of summing up," but it introduced so many difficulties and complications as to lead Mr. Plowden to say, "The matter is all very perplexing. There seem to have been two verdicts, two summings up, and one jury. (Laughter.) All the ordinary landmarks in a court of justice are absent. I have seen something like it on the Savoy stage. (Loud laughter.)" It is, we repeat, the irregularities and "eccentric proceedings," as Mr. Plowden called them, which take place in coroners' courts which lead to the widely-expressed dissatisfaction at "Crowners' quest law."

